# Enhancement of sandy soil water retention using superabsorbent carboxymethyl cellulose grafted with polyacrylamide and polyacrylamidomethyl propanesulfonic acid copolymer

**DOI:** 10.1038/s41598-025-94490-4

**Published:** 2025-05-13

**Authors:** Ahmed A. Abdelgelil, Ahmed M. Omer, Asaad F. Hassan, Ahmed A. Moustafa, Mohamed S. Mohy Eldin

**Affiliations:** 1 Chemistry Department, Faculty of Science, Damnhour University, Damnhour, 22511 Egypt; 2 Polymer Materials Research Department, Advanced Technology and New Materials Research Institute (ATNMRI), City of Scientific Research and Technological Applications (SRTA-City), New Borg El-Arab City, P. O. Box: 21934, Alexandria, Egypt; 3 Polymer Institute of the Slovak Academy of Sciences, Dúbravská Cesta 9, 845 41 Bratislava, Slovakia

**Keywords:** Carboxymethyl cellulose, PAM, PAMPS, SAHs, Grafting, Water uptake, Sandy soil, Chemistry, Materials science

## Abstract

To enhance the productivity of sandy soil, considerable efforts have been devoted to improving its water retention capacity, thereby reducing the frequency of irrigation and minimizing water loss through evaporation. The present study aimed to develop carboxymethyl cellulose (CMC)—grafted-(polyacrylamide (PAM)-co-2-acrylamido-2-methylpropanesulfonic acid (PAMPS) superabsorbent hydrogel (SAH) for effective water retentionin sandy soil. Characterization of the grafted copolymer hydrogel was performed using Fourier transform infrared spectroscopy (FTIR), X-ray diffractometer (XRD), scanning electron microscopy (SEM), and thermogravimetric analysis (TGA). The synthesized CMC-g-(PAM-co-PAMPS) SAH exhibited improved thermal stability, demonstrating a half-weight loss at 391 °C compared to 331 °C for the pure CMC biopolymer. The consequence of various grafting conditions on the percentage add-on was systematically optimized. Additionally, factors influencing water uptake behavior, including contact time, pH and temperature of the medium, particle sizes, and total dissolved salts, were investigated. The results indicated that increasing the co-monomer ratio from 3 to 18% significantly raised the % add-on value from 81 to 97.4%. The developed SAH showed an exceptionalwater uptake capacity of 313 g/g within a short duration of 15 min. Furthermore, it demonstrated the ability to reabsorb water over five successive cycles, achieving an efficiency exceeding 70%. The incorporation of the SAH into sandy soil resulted in a reduction of water outflow, with a significant decrease in the flow rate from 0.96 to 0.32 cm/min. The fabricated superabsorbent hydrogel presents a promising approach for enhancing water retention in sandy soil.

## Introduction

Considering the current global water shortage, agriculture remains one of the largest consumers of water^1^. The poor water uptake capacity and significant deep percolation losses, particularly in sandy soils due to their coarse texture, are critical factors limiting agricultural productivity. Consequently, effective water-soil management strategies must focus on enhancing water retention^2,3^. The use of water-holding amendments, such as polymeric hydrogels, represents a significant advancement in agricultural and landscaping practices, particularly in arid regions^4,5^. Among these hydrogels, superabsorbent hydrogels (SAHs), which are three-dimensional networks constructed from hydrophilic polymers, can absorb water up to thousands of times their dry weight in a short time while retaining it under pressure^6–8^. SAHs not only improve the water-holding capacity of sandy soils but also enhance their physical characteristics and reduce evaporation losses^9^.

Natural polysaccharides such as cellulose, alginate, guar gum, chitosan, and starch are regarded as excellent candidates for creating SAHs due to their superior biocompatibility, biodegradability, low cost, eco-friendly nature, and availability^10,11^. Amongst these, carboxymethyl cellulose (CMC), an anionic water-soluble cellulose derivative produced via the reaction of cellulose with monochloroacetate under alkaline conditions, has been effectively employed across various fields including drug delivery, water treatment, food packaging, detergents, textiles, and paper products^12–14^. Moreover, CMC has the potential to create different kinds of SAHs because of its hydrophilic hydroxyl and carboxylic groups, which can absorb water and moisture^15^. However, native CMC exhibits weak mechanical properties and limited water uptake performance, which can be improved through physical and chemical modifications such as crosslinking and grafting with synthetic acrylate monomers^16–18^. The grafting technique is considered one of the most effective approaches for fabricating superabsorbent hydrogel-based CMC^19^.

Acrylamide (AM), ahydrophilic acrylate derivative, is widely used for the creation of polyacrylamide (PAM) hydrogel^20^. PAM hydrogel has been utilized in the agriculture fieldsdue to its hydrophilic characteristic groups (NH_2_ and OH groups), where the anionic form of PAM is commonly used as a soil conditioner on farmland and construction sites for erosion control^21,22^. The use of PAM in sandy soils has potential not only to improve crop production but also to decrease percolation and evaporation losses of irrigation water^23^. Moreover, PAM hydrogel hasa potential use for controlled release of fertilizerstopromoteplant growth^24^. Additionally, 2-Acrylamido-2-methylpropanesulfonic acid (AMPS) is a multi-group anionic amide monomer, which can be used for the synthesis of poly (AMPS) hydrogel. The sulfonic acid group in AMPS provides hydrophilicity, strong anionic character, exceptional salt resistance, and water absorption capacity to the hydrogel, while the acrylamide group allows for polymerization and crosslinking^25,26^. Because the AMPS products are very affordable, they have grown in importance in the superabsorbent industry^27^. Poly (AMPS) has been widely used in a variety of applications including the production of textiles, personal care products, water purification and agriculture^28,29^. The incorporation of AMPS into hydrogels is a promising approach to improve water use efficiency and nutrient management in agriculture, particularly in arid and semi-arid regions facing water scarcity^30^.

The use of grafted SAHs as conditioners for sandy soils presents several research gaps that warrant further investigation. While existing literature demonstrates the potential of SAHsto enhance water retention, there is a notable lack of comprehensive studies focusing on the optimization of grafting technique to achieve rapid water uptake performance^31–33^. Specifically, many studies have not simultaneously addressed the water retention of the grafted SAHs under varying environmental conditions, which are critical for practical agricultural applications. Furthermore, while some research has explored the mechanical properties of these hydrogels, there is limited understanding of how these properties correlate with their water uptake capabilities over extended periods and under repeated wetting and drying cycles. Addressing these gaps will not only enhance our understanding of grafted hydrogel performance but also inform the development of more effective soil conditioners tailored for diverse agricultural contexts.

The objective of this work was to develop an efficient superabsorbent grafted copolymer hydrogel with fast water uptake performance aimed at improving the water-holding capacity of sandy soil. Two types of hydrophilic acrylate monomers—acrylamide (AM) and 2-acrylamido-2-methylpropanesulfonic acid (AMPS)—were grafted onto a CMC backbone via free radical polymerization. Factors affecting the grafting process were studied and optimized. The impacts of contact time, temperature, pH, total dissolved salts, and particle size on the water uptake profiles of the developed SAH were investigated. Additionally, weight loss after re-swelling for several cycles was examined. Finally, the ability of sandy soil amended with hydrogel to retain water and the effect of SAH on water flow rate were evaluated.

## Experimental

### Materials

Carboxymethyl cellulose sodium salt, (CMC; DS = 0.7; Mw. 90,000), 2-Acrylamido-2-methylpropanesulfonic acid (AMPS; assay 99%; Mw. 207.25), ammonium persulfate (APS; assay > 99%; Mw. 228.2) and N, N´-methylene bisacrylamide (MBA; assay > 99%;Mw. 154.17)were purchased from Sigma–Aldrich chemicals ltd. (Germany). Polyethylene glycol (PEG; Mw. 950–1050) was acquired from PARK scientific limited wothampton(UK). Acrylamide (AM; assay > 98%; Mw. 71.08), wasprocured from Alpha Chemika, (India). Sodium bicarbonate (NaHCO_3_; Mw.84), Sodium hydroxide (NaOH; assay 99%; Mw. 40), Hydrochloric Acid (HCl; assay 37%; Mw. 36.47) and Acetone, (purity 99%; Mw. 58.08) were obtained from EL-Nasr pharmaceutical Co. (Egypt). Sodium carbonate(Na_2_CO_3_; assay 99.5% Mw. 105.99)wasbought from Rankem Co.(India). Sandy soil was collected from desert of Alexandria, Egypt.

### Preparation of the SAH

**Fig. 1:**
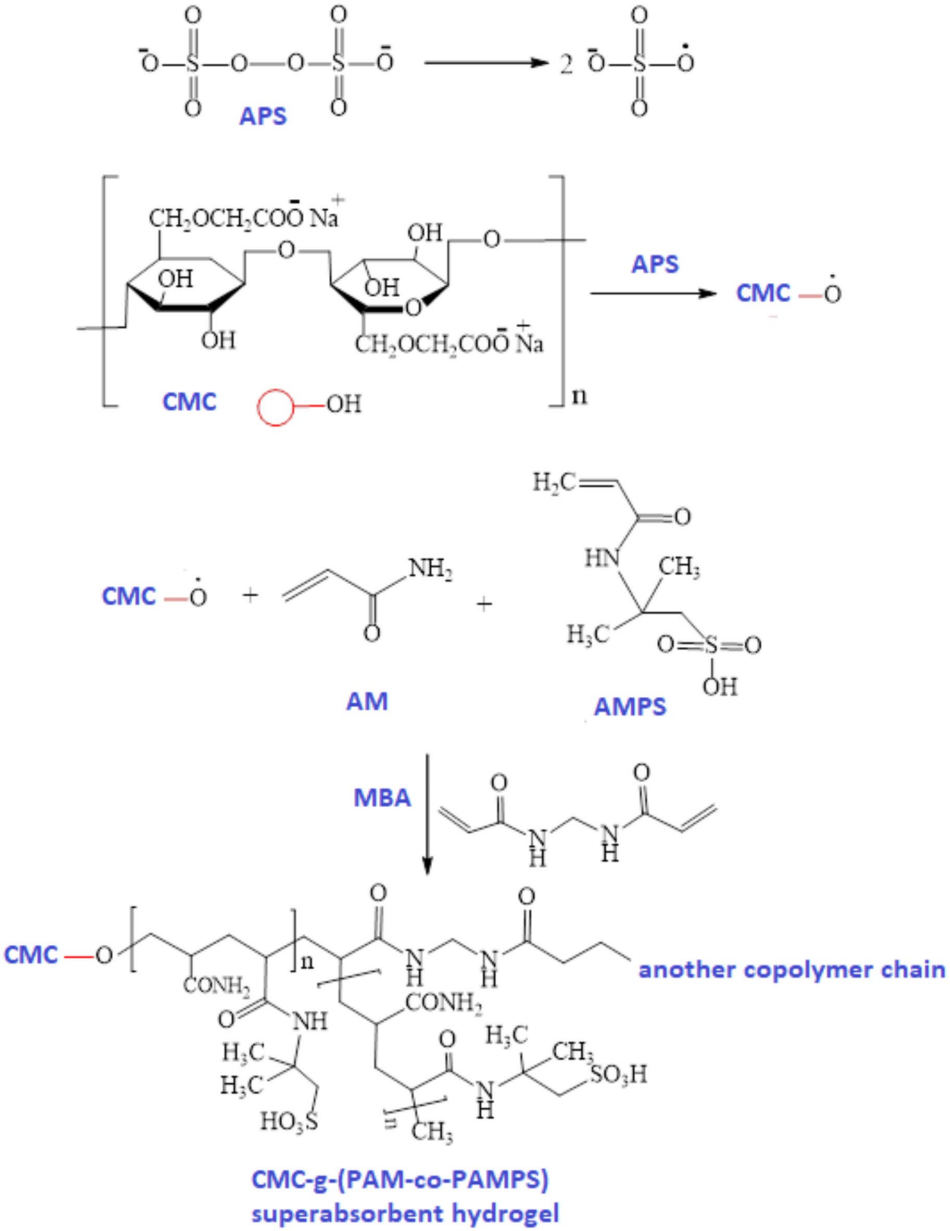
Synthesis ofCMC-g-(PAM-co-PAMPS) SAH.

The free radical polymerization approach was used to create a variety of CMC-g-(PAM-co-PAMPS) SAHs^34^. Briefly, hot distilled water (60 °C) was used to dissolve CMC(0.5–3%; w/v) under a continuous stirring. Subsequently, PEG (0.067%; w/v) was added to the CMC solution. APS (0.05–0.3%; w/v) was added to the reaction solution under stirring for 15 min to generate radicals on the CMC backbone. Next, AM and AMPS were dissolved in distilled water before being added to the reaction mixture. Thetotal concentration ofboth AM and AMPS were adjusted at3, 6, 9, and18%; w/v, while the AM: AMPSratioswere2.5:0.5, 2:1, and 1.5:1.5, respectively. Eventually, sodium bicarbonate (0.04%; w/v), sodium carbonate (0.067%; w/v) and crosslinker MBA (0.056–0.283%; w/v) were added to the grafting mediumunder continuous stirring. The grafting process was carried out in a water bath at various polymerization temperatures (40–80 °C), and the grafting time was investigated between 0.5 and 5 h. The created hydrogel was allowed to sit overnight (post-grafting), followed with washing several times using hot distilled water to eliminate the residual unreacted monomers and homopolymers. Then, the grafted hydrogel was dried in an oven at 60 °C for 24 h until a constant weight was achieved. The dry gel (xerogel) was crushed and passed through sieve shakers to gain various particle sizes (125 µm–1 mm). The synthesis mechanism ofCMC-g-(PAM-co-PAMPS) SAHwas depicted in Fig. 1. The grafting process was assessed using % polymer add-on according to the following equation:

1 $$\% {\text{Add - on = }}\left( {\frac{{{\text{W}}_{2} {\text{ - W}}_{1} }}{{{\text{W}}_{2} }}} \right)$$

where ${W}_{1}$ and ${W}_{2}$ are the weights of CMC and grafted copolymer hydrogel, respectively.

### Characterization

Using the Fourier Transform Infrared (FTIR) spectra (Shimadzu FTIR—8400 S, Japan), the structure of the created hydrogel was investigated. X-ray diffractometer (XRD; MAC Science M03XHF) was used to explore the crystallographic structure, while Thermo gravimetric analyzer (Perkin–Elmer Cetus Instruments, Norwalk, CT) was employed to examine the thermal properties. Moreover, the morphological propertieswere inspected by a Scanning Electron Microscopy (SEM; Joel-Jsm 6360LA, Japan).

### Water uptakestudies

The water uptake capacity of the developed superabsorbent hydrogels (SAHs) was assessed based on methodologies established in the author’s previous research^35^. Specifically, a precise amount of 0.1 g of dried sample was submerged in 25 mL of waterfor a definite time period. Thereafter, the swelled samples were carefully separated from the swelling medium and placed between two filter papers to eliminate any excess water adhered on the surface of samples. Following this process, the samples were weighed immediately using an electronic scale to ensure accuracy in measurement. The experimental design incorporated variations in sample particle size (125 µm to 1 mm), swelling time (2–60 min), medium temperature (25 °C–50 °C), and swelling medium pH (3–8) to assess their impact on the water uptake profile; pH was adjusted using 0.1 N HCl and 0.1 N NaOH solutions.

Additionally, the study investigated the impact of total dissolved solids (TDS) on water uptake profiles by comparing tap water (TDS: 375 ppm, pH: 7.4, conductivity: 914 μS) and distilled water (TDS: 1.2 ppm, pH: 7.1, conductivity: 3.58 μS). The water uptake (WU) was determined using the following equation:

2 $$\text{WU }(\text{g}/\text{g}) =\left(\frac{{\text{W}}_{\text{t}}-{\text{W}}_{\text{i}}}{{\text{W}}_{\text{i}}}\right)$$

where ${W}_{i}$ and ${W}_{t}$ are the weights of the initial dried samples and the swollen SAH in the time of measuring (t), respectively.

### Reuptake and re-drying studies

The reuptake of water and re-drying profile of CMC-g-(PAM-co-PAMPS) were investigated through five successive cycles. In each cycle, the dried sample was immersed in distilled water for 15 min to allow for swelling, after which the swollen hydrogel was weighed. Subsequently, the SAH was dried at 60 °C for 24 h until a constant weight was achieved. The water uptake (g/g) and the percentage of the initial dried sample weight remaining after each cycle were then calculated using Eq. (2).

### Water uptakeand flow rate in sandy soil

To evaluate the water conservation capabilities of the synthesized superabsorbent hydrogel (SAH) within sandy soil, a controlled experiment was conducted using two identical plastic measuring cylinders (1000 ml volume, 40 cm height, 39.57 cm^2^ area). Both cylinders were modified to include small, uniform perforations at the base, over which a filter paper was placed. The first cylinder contained a homogenous mixture of 5 g of dried SAH and 495 g of sandy soil of comparable particle size ranged from 250 to 500 µm, resulting in a total height of 10 cm. The second cylinder, serving as a control, was filled with 500 g of pure sandy soil to the same height. Both cylinders were positioned atop plastic beakers to collect drained water. Tap water was continuously introduced into both cylinders for 5 h to ensure complete saturation of the SAH within the sandy soil matrix. The experiment was replicated with a total sandy soil height of 20 cm to assess the effect of soil depth on the water uptake profile. Water uptake by the SAH was determined by measuring the weight differential between the SAH-amended sandy soil and the pure sandy soil. The volume of water outflow (ml) and the corresponding water uptake (g/g) were measured concurrently. The water flow rate was then calculated according to the following equation^24^:

3 $$\text{Flow rate }(\text{cm}/\text{min})=\left(\frac{\text{Volume of Flow Water }({\text{cm}}^{3} )}{{\text{Area of Cylinder }\left({\text{cm}}^{2}\right)\times \text{ Time of Flowing }\left(\text{min}\right)}}\right)$$

All of the grafting and water uptake experiments were performed in triplicate, and the data were represented as mean standard deviation (± SD).

## Results and discussion

### FTIR spectra

**Fig. 2:**
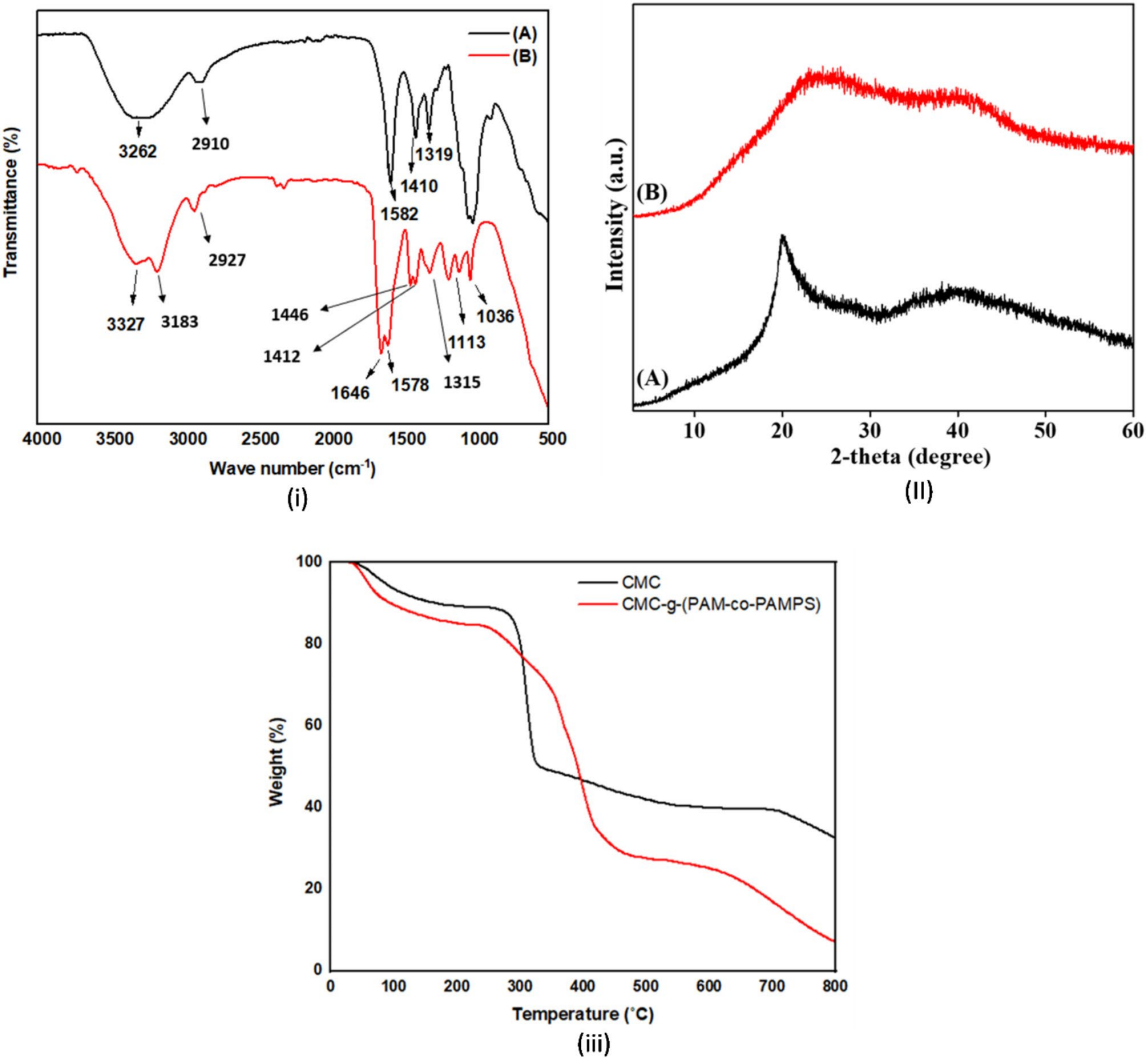
(**i**) FTIR spectra, (**ii**) XRD patterns, and (**iii**) TGA curves of CMC and its grafted copolymer hydrogel.

FTIR spectra of CMC and grafted copolymer hydrogel were portrayed in Fig. 2(i), respectively. According to Fig. 2(i)A, The stretching frequency of CMC’s hydroxyl groups is responsible for the observed absorption peak at 3262 cm^−1^ and peak at 2910 cm^−1^ is due to C–H stretching vibration^36^. A significant absorption peak at 1582 cm^−1^ was caused by the existence of carboxylate groups. The peaks near 1410 and 1319 cm^−1^ were attributed to CH_2_ scissoring and vibration of -OH bending, successively^37^. In the case of CMC-g- (PAM-co-PAMPS), a broad absorption band at 3327 cm^−1^ was attributed to the overlap of hydroxyl groups of CMC with the amino groups of the copolymer. In addition, peaks around 3183 and 2927 cm^−1^ associated with the C-H stretching frequency of –CH_3_ and –CH_2_ groups and other peaks at 1315and 1412 cm^−1^ are attributed to CH_2_ scissoring and CH_2_ twisting(Fig. 2(i)B)^19,38^. The stretching frequency of 1646, 1446 and1036 cm^−1^ were attributed to NH bending (Amide II band), C–N stretching of secondary amide (–CONHR) and CH–O–CH_2_ group, respectively. The detected peaks at 1113 and 1578 cm^−1^ were attributed to the sulfonate groupsin AMPS unite and the C=O stretching mode of the amide groups of AM^39^, confirming that the graft copolymerization has taken place onto the CMC chains as evidenced by the obvious alterations in the chemical structure of CMC.

### XRD analysis

The crystallographic patterns of pure CMC and CMC-g-(PAM-co-PAMPS) SAH provide insights into the microstructural changes induced by the grafting process and their correlation with swelling behavior. The pristine CMC (Fig. 2(ii)A) exhibits a broad peak at a 2-theta value of 19.90º, indicating its low crystalline nature. This crystalline structure is disrupted in the CMC-g-(PAM-co-PAMPS) SAH (Fig. 2(ii)B), as evidenced by the disappearance of the characteristic peak and the emergence of an amorphous pattern. This transformation signifies successful grafting of PAM and PAMPS onto the CMC backbone, which fundamentally alters its microstructure and enhances its water uptake performance. The amorphous nature observed in the grafted hydrogel facilitates higher water uptake due to increased free volume and reduced structural constraints, enabling water molecules to penetrate more easily into the polymer network. Studies have shown that amorphous regions in hydrogels provide greater flexibility to polymer chains, allowing them to expand and accommodate water molecules more effectively^40^. Additionally, the sulfonic acid groups from AMPS and amide groups from PAM introduce strong hydrophilic interactions that enhance water absorption through hydrogen bonding and osmotic pressure gradients. These microstructural modifications make the grafted SAH highly effective for rapid water uptake and retention, particularly when applied as a soil conditioner in sandy soils where efficient moisture management is critical for agricultural productivity.

## Thermal stability analysis

Figure 2(iii) and Table 1 showed the impact of the grafting process on the thermal stability of the fabricated SAH. At the ambient temperature (0–120 °C), the observed first weight loss was caused by the absorbed humidity in the investigated materials, which was 7.8% for CMC and 11.6% for the grafted copolymer hydrogel^41^. The slight increase in the weight loss in the grafted copolymer could be ascribed to the present more hydrophilic groups. With increasing temperature from 200 to 400 °C, the grafted copolymer hydrogeldemonstrated greater thermal stability, since the rate of weight loss was significantly lower than pure CMC biopolymer. This might be attributed to the cross-linked matrix formation that was created by the graft copolymerization process. The measured weight losses may be related to the decarboxylation of CMC, the decomposition of the saccharide rings, and the elimination of CO_2_ from the polymeric chains^19,36^. These results were verified by determining the temperature at which materials must lose half their weight (T_50%_°C). The grafted sample showed a higher T_50%_°C value of 391 °C while pure CMC was 331 °C, demonstrating the good thermal stability of the hydrogel made of CMC-g- (PAM-co-PAMPS) hydrogel.

**Table 1:** TGA data for pure CMC and CMC-g-(PAM-co-PAMPS) SAH.

Sample	Weight loss (%)		T_50%_ °C
	0–120 °C	400 °C	
CMC	7.8	53.39	331
CMC-g- (PAM-co-PAMPS)	11.6	51.63	391

### SEM analysis

**Fig. 3:**
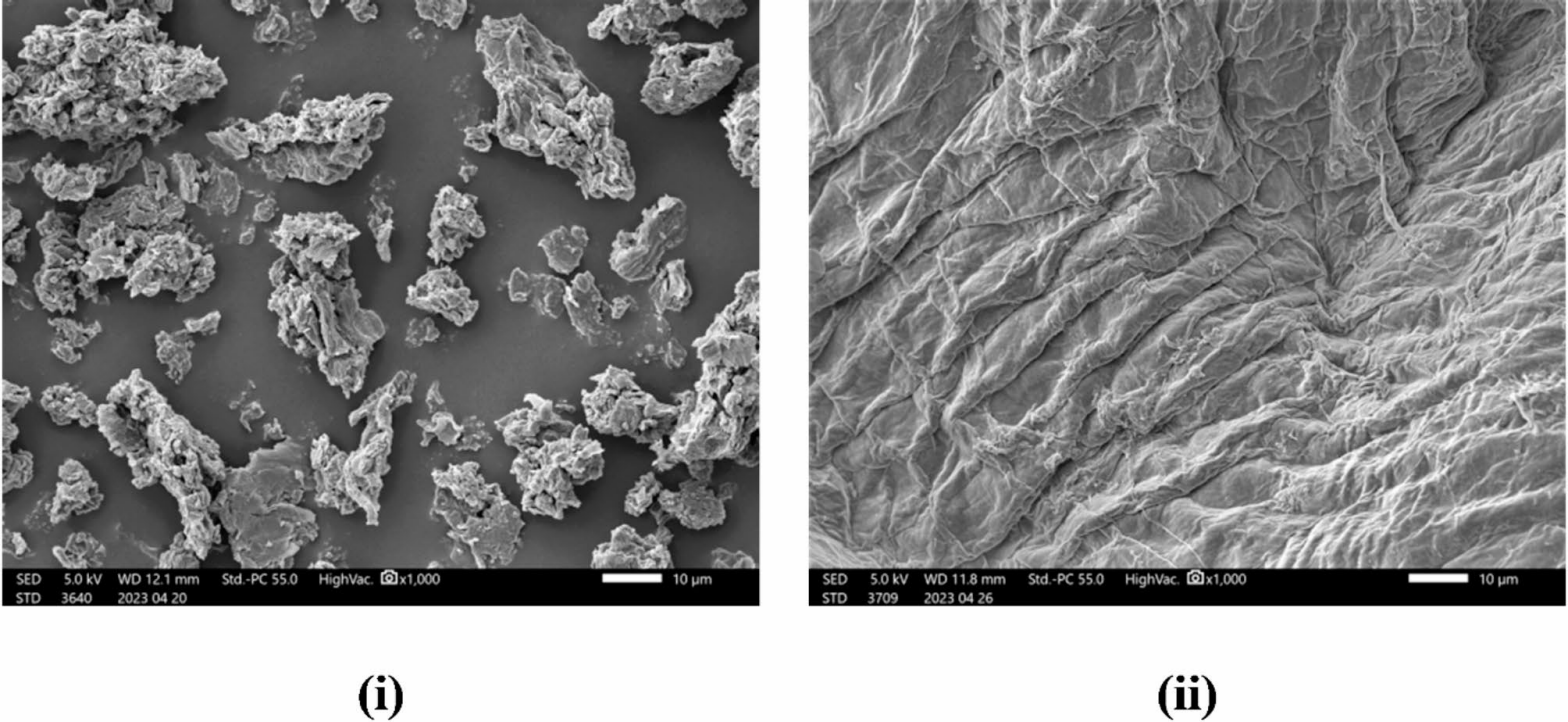
SEM images of **(i)** CMC and **(ii)** CMC-g- (PAM-co-PAMPS) SAH.

The morphological alterations of the pure CMC and its grafted copolymeric hydrogel were examined by SEM analysis. As seen in Fig. 3(i), the surface of CMC displayed a granular structure with large and irregular particles^42^. After the grafting process, itwas fully transformed to rough, wrinkled and undulating surface as depicted in Fig. 3(ii). These changes could be a result of the graft copolymerization of PAM and PAMPS along the CMC backbone, in addition to polarity difference between the hydrogel compositions^43^.

### Impacts of the grafting process on % polymer add-on and water uptake profiles

#### Impact of CMCconcentration

**Fig. 4:**
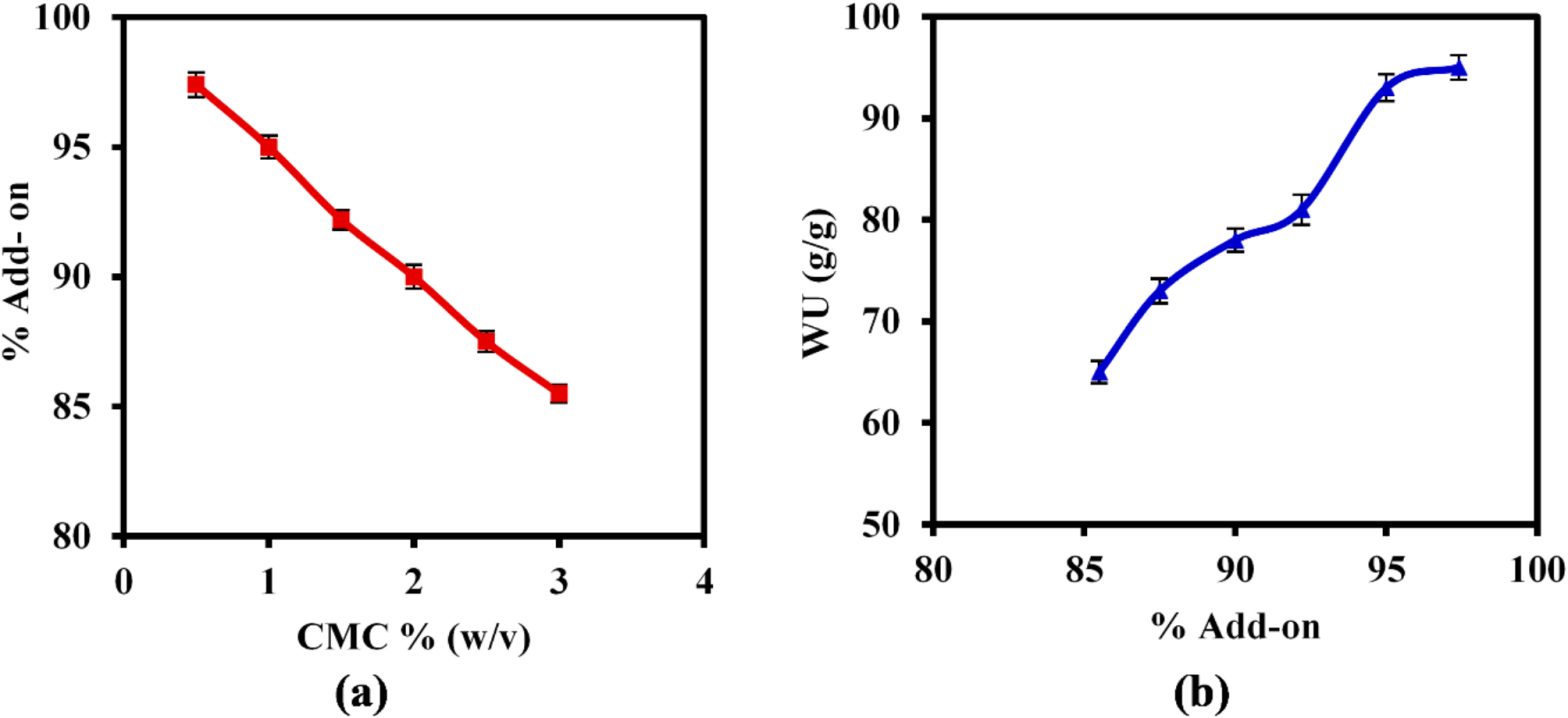
(**a**) Effect of CMC variation on the% Add-onvalues, and (**b**) Effect of % Add-onon the WU(g/g) values of CMC-g- (PAM-co-PAMPS) SAH.

The impact of variations in CMC concentration on the (%) add-on and water uptake (WU) of the superabsorbent hydrogel (SAH) was investigated, as illustrated in Fig. 4. An increase in CMC concentration from 0.5 to 3% (w/v) resulted in a significant decrease in the (%) add-on, from 97.4 to 85.5% (Fig. 4(i)). This phenomenon can be attributed to the significant increase in viscosity of the grafting mixture. As CMC concentration rises, the viscosity escalates, which in turn restricts the mobility of monomers towards the reactive sites on the CMC chains. This impediment results in fewer monomers being able to effectively attach to the CMC backbone, leading to a lower % add-on^19^. Conversely, at lower concentrations of CMC, there is greater mobility of CMC chains and monomers, facilitating better grafting and potentially increasing % add-on values.

Besides, the WU valueincreased from 65 to 95 g/g with increasing the % add-on from 85.5 to 97.4% as displayed in Fig. 4(ii). These results could be attributed to increasing the number of hydrophilic groups (NH_2_, SO_3_H, OH) with increasing the number of grafted acrylate monomers, improving the hydrophilicity of the hydrogel network^44^. Thus, the affinity of water molecules to diffuse into the hydrogel matrix increases, resulting in a greater WU values.

#### Impact of the total co-monomers quantity and their ratios

**Fig. 5:**
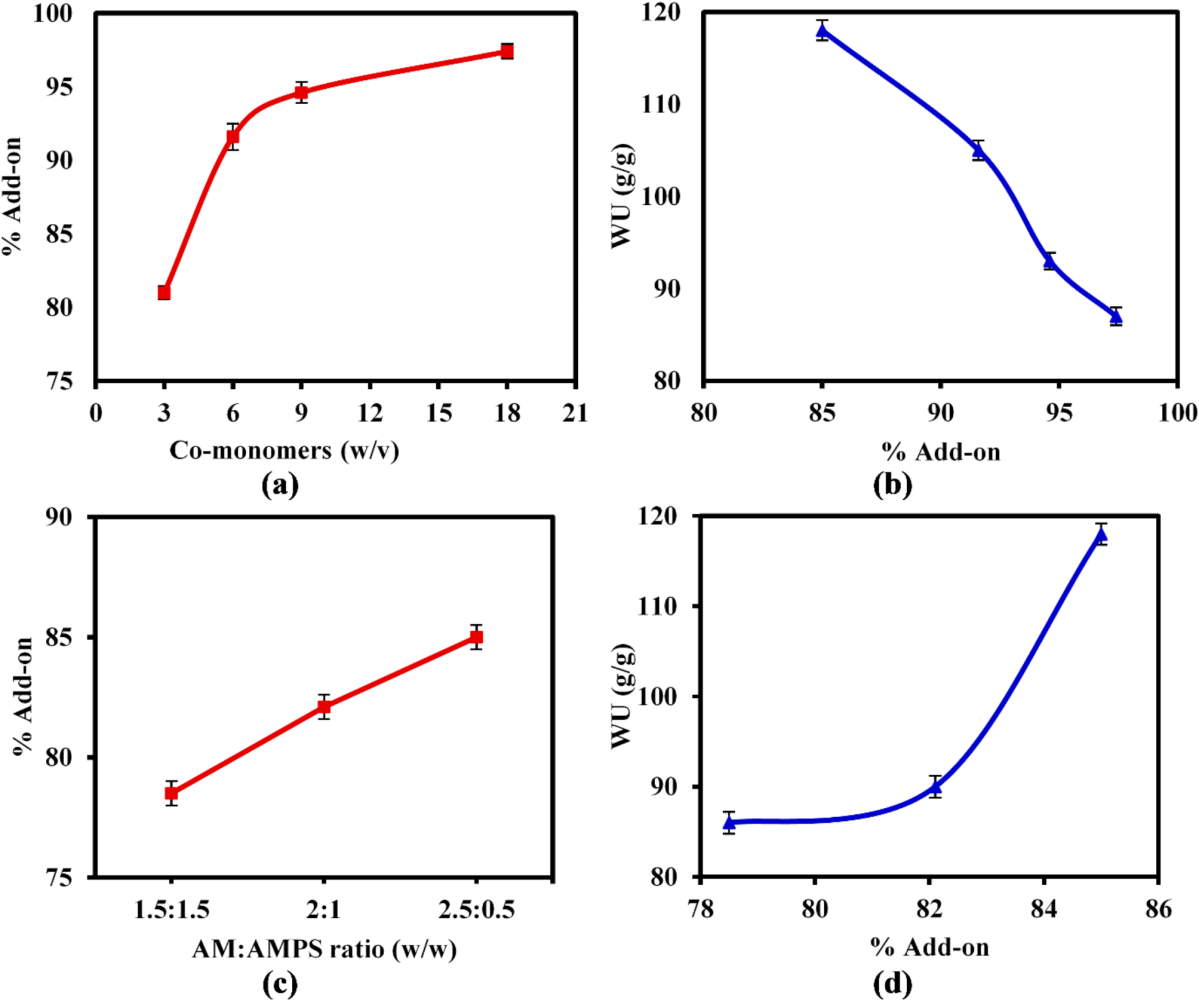
Effect of total co-monomers amount and their ratios on(**a, c**) the % Add-on, and (**b**, **d**) WU (g/g) values of the SAH.

The effect of the total amount of co-monomers (AM and AMPS) on the percentage of add-on values was examined within the range of 3–18% (w/v), as illustrated in Fig. 5a. It is evident that the (%) add-on value increased with the total quantity of monomers, with the highest recorded value being 97.4% at a concentration of 18% (w/v)^45^. This might be explained by the fact that raising the total monomer amount significantly encourages their diffusion toward the immobile CMC macroradicals. The enhanced availability of co-monomers within the polymerization medium promotes molecular collisions, thereby accelerating the graft copolymerization process and resulting in an increase in the (%) add-on value. On the other side, the lowest co-monomers amount recorded the highest WU value as presented in Fig. 5b. Indeed, raising the co-monomers amount increases the viscosity of the grafting medium, and as a result, a highly dense networked hydrogel produces. This in turn reduces the amount of penetrated water molecules into the hydrogel network, resulting in a decrease in the WU value from 118 to 87 g/g with increasing the total amount of co-monomers from 3% (85% add-on) to 18% (97.4% add-on).

Additionally, the impact of varying AM:AMPS ratios on the % add-on was investigated, as illustrated in Fig. 5c. The results obtained elucidated that a higher ratio of AM in the co-monomers facilitated the grafting process, resulting in an increase in the % add-on to 85% when employing a 2.5 (AM): 0.5 (AMPS) ratio, in contrast to a decrease to 78.5% observed with a 1.5 (AM): 1.5 (AMPS) ratio^46^. The AM monomers exhibit greater reactivity compared to the AMPS monomers, attributable to the presence of an amide group that readily forms polymer chains. Furthermore, AM demonstrates greater tolerance for radical polymerization conditions, leading to enhanced grafting efficiency and a more robust attachment of the polymer chains to the CMC backbone, thus yielding a higher % add-on value. In contrast, AMPS monomers possess a heightened propensity to undergo homopolymerization rather than copolymerization with the grafted AM monomers on the CMC backbone. Consequently, an increase in the AMPS ratio can impede the initiation and propagation stages of the polymerization process due to the presence of the negatively charged sulfonic acid group in AMPS, which directly leads to chain transfer and termination reactions. This phenomenon can further diminish the efficacy of the graft copolymerization process^47^.

Besides, increasing the ratio of AM was more beneficial in improving water uptake behavior of the developed CMC-g-(PAM-co-PAMPS) SAH. It was observed from Fig. 5d that increasing the ratio of AM monomers in the co-monomer (i.e. 2.5 (AM): 0.5 (AMPS); 85% add-on) enhances the water uptake behavior with a maximal value of 118g/g. These results could be due to that AM is a hydrophilic monomer, which can form hydrogen bonds with water molecules, allowing for increased water absorption capacity. On the other hand, increasing the AMPS ratio may not necessarily result in higher water uptake, since AMPS is a potent polyelectrolyte that can obstruct the formation of hydrogen bonds with water molecules, leading to decreased water absorption capacity of the SAH.

#### Impact of crosslinker (MBA)

**Fig. 6:**
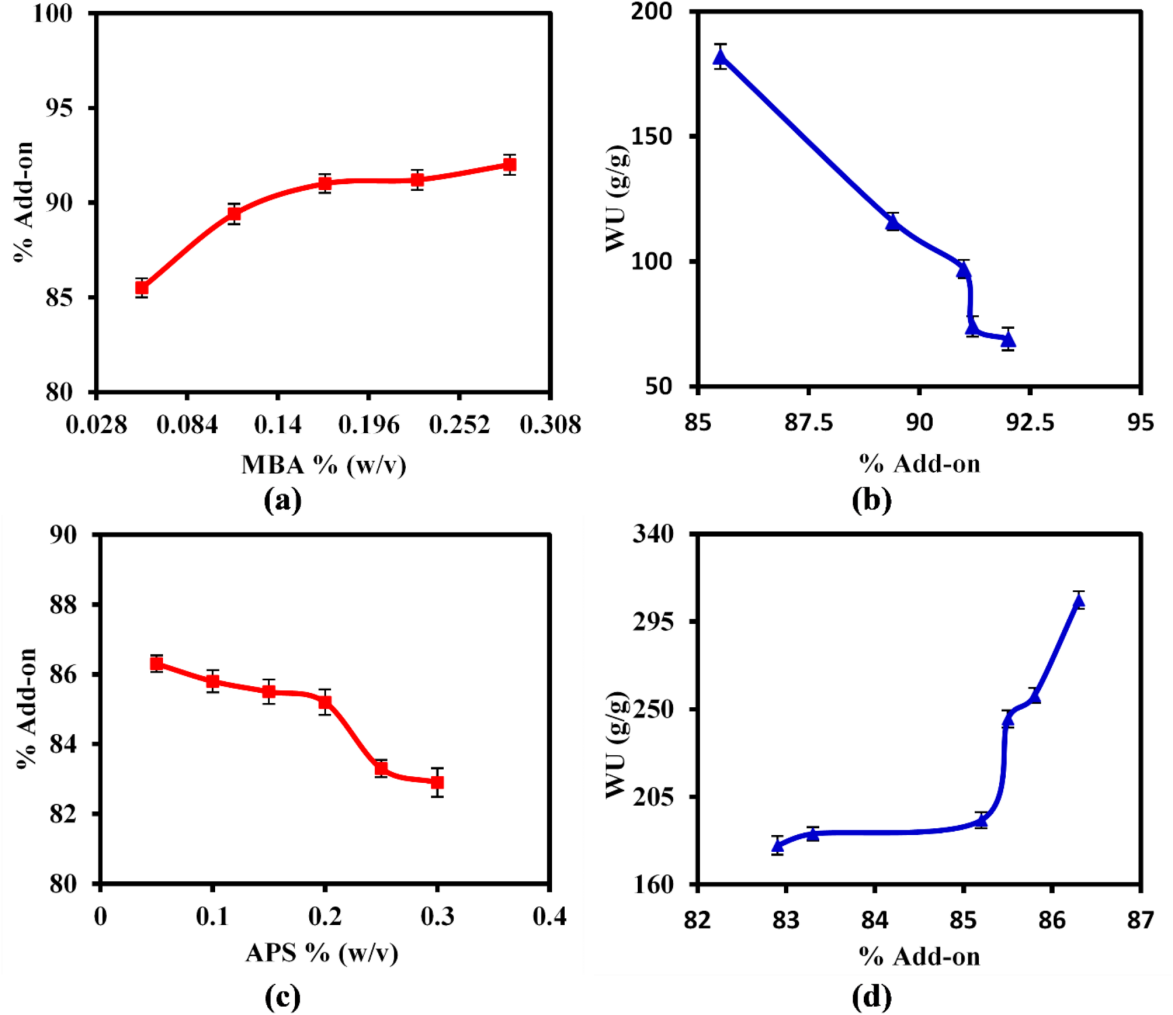
Effect of MBA and APS concentrations on (**a**, **c**) the % Add-on, and (**b**, **d**) WU (g/g) values of the SAH.

The effect of the amount of crosslinking agent (MBA) on the % add-on value was analyzed, as illustrated in Fig. 6a. It was observed that raising the MBA concentration from 0.056 to 0.286% (w/v) resulted in an increase in % add-on from 85.5 to 92%. These results may be due to the enhanced likelihood of co-monomers copolymerizing as the MBA concentration rises, which subsequently increases the crosslinking density of the resulting three-dimensional network structure of the grafted hydrogel, thus raising the add-on value^48^. Nevertheless, Fig. 6b clarified that the hydrogel sample with lower% add-on recorded the maximal WU value of 183 g/g, and then tend to gradually decrease with increasing of % add-on.

#### Impact of initiator (APS)

Figure 6c shows that increasing the concentration of APS from 0.05 to 0.3% (w/v) resulted in a slight decrease in the % add-on from 86.3 to 82.9%. However, the peak water uptake capacity of 306 g/g was reached with an % add-on value of 86.3% (Fig. 6d). These results may be attributed to the higher concentration of initiators, which promotes the production of numerous free radicals and a greater density of active sites. This can lead to a rapid termination process^19^. As a result, this quick termination may shorten the average length of the polymer chains, which could lead to a decrease in both the add-on percentage and water uptake (WU) values. On the other hand, lowering the initiator concentration encourages the creation of free radicals and active sites due to the breakdown of the APS initiator along the polymer chains^49,50^. This leads to a higher add-on percentage and, subsequently, increased water uptake, as well as a greater amount of grafted hydrophilic monomer chains.

#### Impact of grafting time

**Fig. 7:**
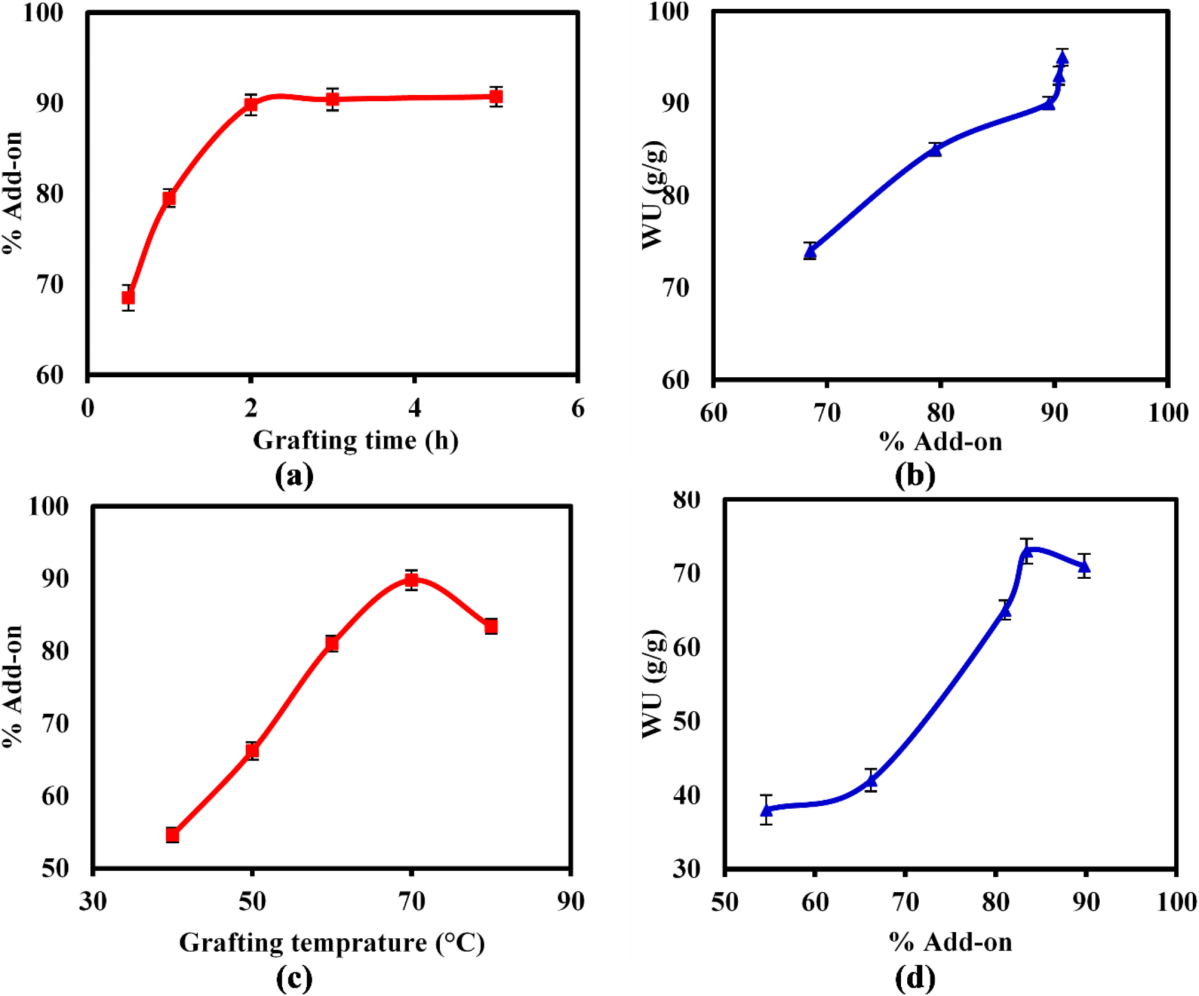
Effect of grafting time and temperature on (**a**, **c**) the % Add-on, and (**b**, **d**) WU (g/g) values of the SAH.

Figure 7a signified that the % add-on on increased from 68.5% to a maximum of 89.8% as the grafting time was extended from 0.5 to 2 h. This augmentation in grafting time facilitates a greater opportunity for the AM and AMPS monomers to diffuse and react with a larger number of initiated active sites on CMC backbone, resulting in a higher degree of grafting and an increased % add-on. However, the percentage of add-on did not exhibit further increase even with an extension of the grafting time to 5 h, suggesting that the majority of available active sites had already been utilized^51^. Accordingly, the grafting process has reached equilibrium, and the percentage of add-on remains relatively constant. Likewise, Fig. 7b demonstrates that as the percentage of add-on increases over 2 h, the hydrophilic nature of the grafted copolymeric hydrogel also enhances, thereby improving its water absorption capacity and resulting in a higher water uptake (WU) value.

#### Impact ofgrafting temperature

Various temperatures ranging from 40 to 80 °C were employed to examine their impact on the % add-on and water uptake profiles of the produced hydrogel. As illustrated in Fig. 7c, the % add-on increased from 54.6 to 89.8% when the grafting temperature rose from 40 to 70 °C, but then began to decrease with further temperature increases up to 80 °C. These findings may be attributed to the heightened rate of APS decomposition and the increased reactivity of monomers at elevated temperatures^52,53^. This leads to the formation of a higher number of active radical sites on the CMC backbone, facilitating the grafting of an increased quantity of monomers and resulting in a higher percentage of add-on. Conversely, the observed decrease in the % add-on at temperatures exceeding 70 °C may be attributed to the accelerated termination of the grafting process at elevated temperatures, which significantly impedes the effectiveness of the grafting process. Similarly, Fig. 7d demonstrates that the WU value increases with the rise in percentage add-on, due to the incorporation of a greater number of hydrophilic groups into the hydrogel structure. Consequently, this enhances the hydrophilicity of the resultant hydrogel network, thereby creating additional binding sites for water molecules^54^.

### Factors affecting water uptake profile

The impact of adjusting operating parameters, such as swelling time, pH, temperature of aqueous mediumand hydrogels’ particle size, total dissolved saltson the water uptake behavior has been studied as presented in the following sub-sections.

#### Effect of contact time

**Fig. 8:**
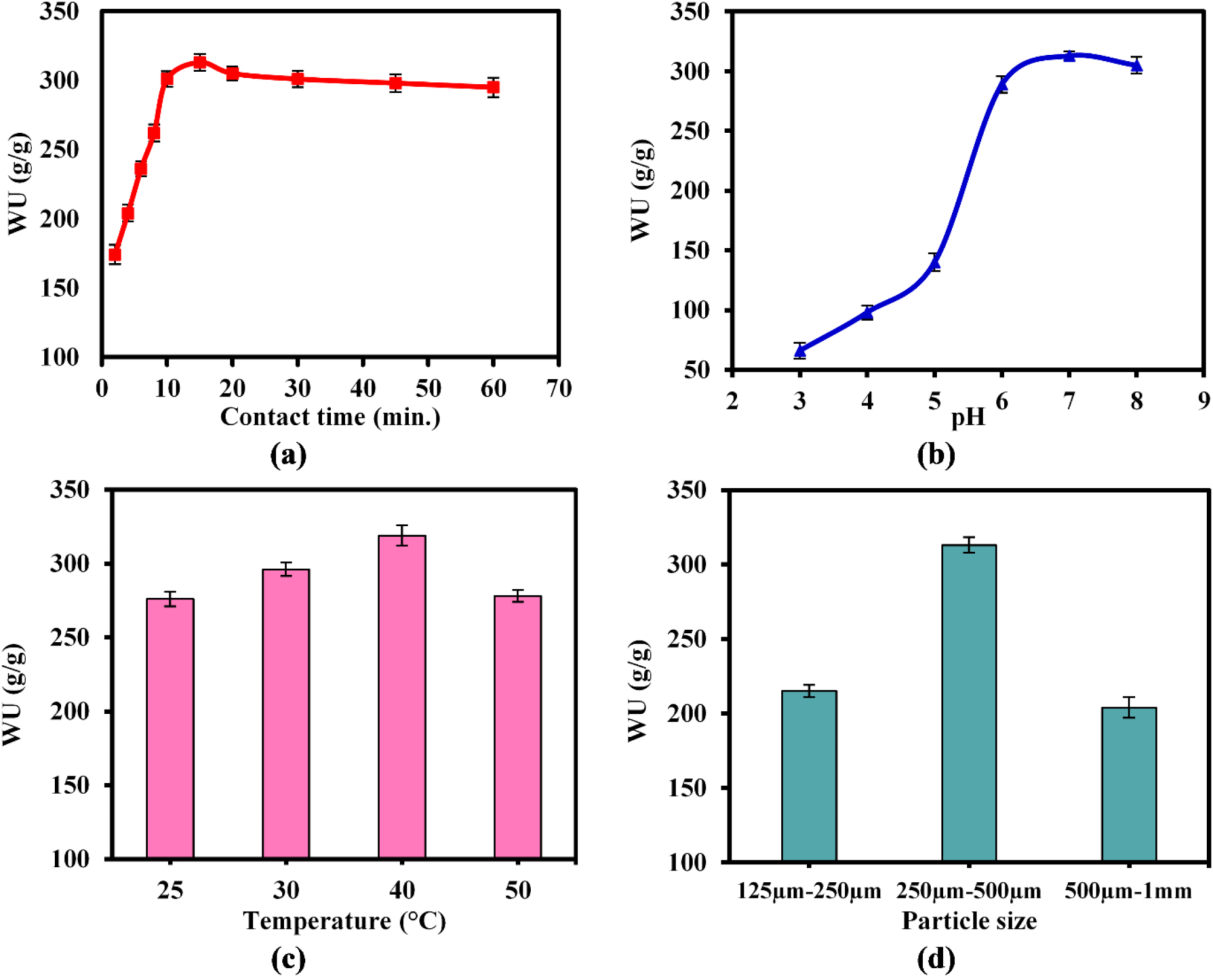
Effect of (**a**) contact time, (**b**) pH medium, (**c**) medium temperature, and (**d**) hydrogel particle size on the water uptake of SAH.

The water uptake behavior of the created CMC-g-(PAM-co-PAMPS) SAH was investigated at various swelling times through 60 min as shown in Fig. 8a. Observations revealed that the WU values exhibited a rapid increase within the initial 10 min, thereafter attaining equilibrium after 15 min with a highest value of 313 g/g. Subsequently, the water uptake capacities were quite stable as the time increased up to 60 min. When the hydrogel is placed in water, the hydrophilic groups in the hydrogel matrix rapidly absorb water molecules through hydrogen bonding and electrostatic interaction^55^. This results in a speedy uptake of water by the hydrogel at the beginning. Later, the hydrogel structure attains a state of equilibrium, when the hydrogel network becomes completely hydrated and the rates of water absorption and desorption stabilize^24,56^. Therefore, water equilibrium uptake duration of 15min was chosen for the subsequent studies.

#### Effect of pH medium

The pH of the surrounding environment significantly affects the manner in which the superabsorbent hydrogel absorbs water. As depicted in Fig. 8b, the water uptake value at high pH levels was much greater than that observed at low pH levels. These findings could be explained by the fact that both the sulfonic and carboxylic groups of AMPS and CMC exist in the protonated form in an acidic media (i.e. pH 3–4). Therefore, there was an overall reduction in the repulsive forces and an increase in the hydrogen-bonding interaction, resulting in a higher density of the hydrogel network. So, the sole accountable entities for uptake water are the protonated amino groups of AM (i.e. NH_3_^+^), leading to the attainment of minimal water uptake values at low pH levels of 3–4 (66–98 g/g). Conversely, a substantial rise in the WU value appeared at high pH levels (i.e. pH 6–8) with maximum values ranging from 289 to 313 g/g. This increase can be attributed to the enhanced repulsive interactions between anions (COO^−^ and SO_3_^−^) resulting from their deprotonation^44^. This results in an immediate expansion of the hydrogel network, causing an increase in the WU values. Similar pH-dependent water uptake behaviors have been reported^57,58^.

#### Effect of medium temperature

Figure 8c showed that the WU profile was clearly increased from 276 to 319 g/g with rising the swelling medium temperature from 25 to 40 °C. These results could be ascribed to that increasing medium temperature could increase the hydrogel matrix’s elasticity with enhancing chains mobility and reduce hydrogen bonding^59^. This causes more water molecules to enter the hydrogel matrix, boosting water uptake. Nevertheless, further increasing temperature up to 50 °C, the water uptake value declined to 278g/g as a result of deformation of the hydrogel network and lost its mechanical capabilities^60^.

#### Effect of particle size

The study investigated the influence of particle size variation on the water uptake profile of CMC-g-(PAM-co-PAMPS) SAH. Three distinct ranges of particle size were examined, as shown in Fig. 8d. The findings revealed that the hydrogel sample with particle size ranging from 250µm to 500µm exhibited the greatest water uptake (WU) value of 313g/g, surpassing the sample with larger particle size (500 µm–1 mm). This can be attributed to the increased surface area of the particles, which allows for more water uptake^61^. Even so, the hydrogels with the smallest investigated particle size of 125 µm–250 µm exhibited a modest water uptake (WU) value of 215g/g. This might be attributed to their tendency to agglomerate, which diminishes the accessible surface area for water uptake. Agglomeration can result in a compact structure, while diffusion path length for water molecules to penetrate may be restricted. This can decelerate the rate at which water is absorbed and reduce the total ability for water uptake. Besides that, hydrogels with particles smaller than 125µm lack mechanical stability, causing its network to quickly distort during the experiment. This deformation makes it difficult to handle and correctly measure the water uptake.

#### Effect of total dissolved salts (TDS)

**Fig. 9:**
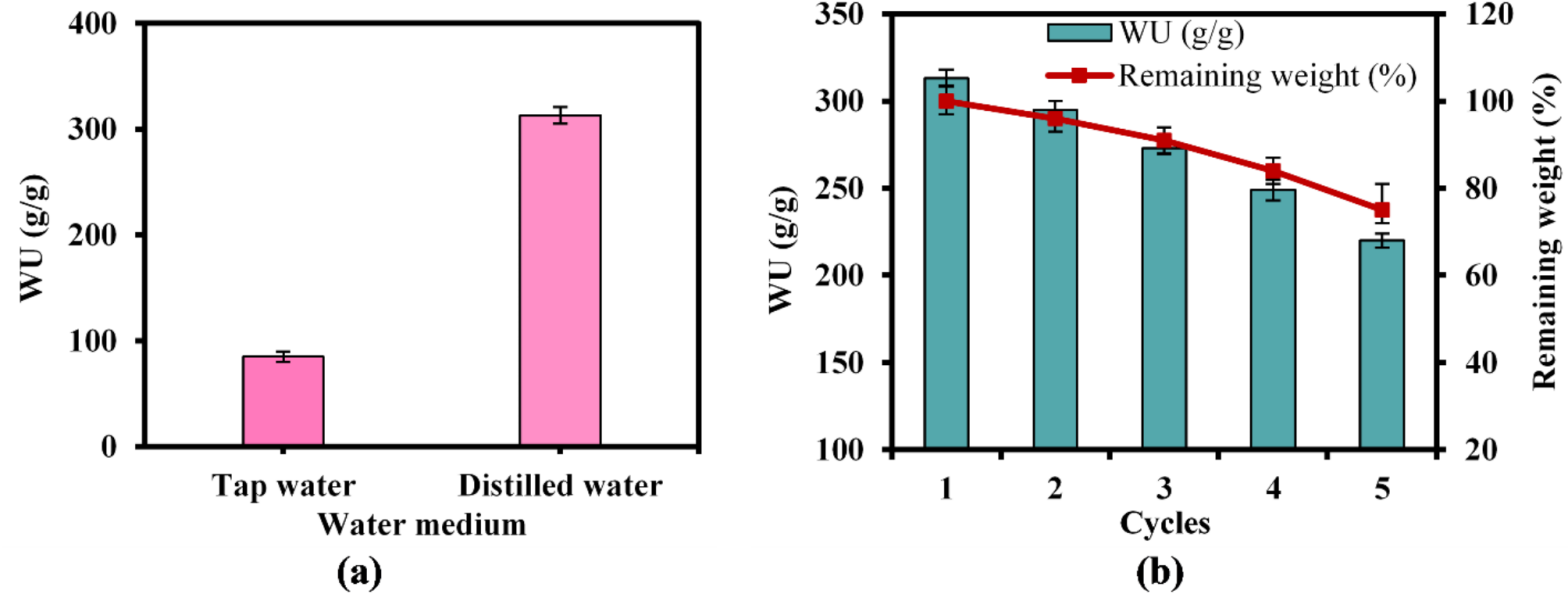
Effect of (**a**) TDS and (**b**) Reuptake and re-drying cycles on the water uptake of CMC-g-(PAM-co-PAMPS) SAH.

To examine the impact of total dissolved salts (TDS) on water uptake profile, distilled water and tap water were used as water uptake media with TDS concentrations of 1.2 and 375 ppm, respectively. Based on the findings presented in Fig. 9a, the water uptake of the developed grafted copolymer hydrogel in tap water was substantially reduced compared to distilled water^62^. The ions that are dissolved in tap water have the potential to interact with the functional groups present in the hydrogel network, which might potentially cause changes in the water uptake capabilities. Hence, the difference in mobile ion concentrations between the tap water and the SAHs results in an osmotic gradient that restricts the absorption of water by the hydrogel. This is because the water molecules must overcome the osmotic pressure differential in order to enter the hydrogel network. Consequently, the volume of the SAHs matrix is reduced, leading to a decrease in its capacity to absorb water^63,64^.

### Effect of reuptakecycles

The mechanical characteristics of the superabsorbent hydrogel affect how much water it can uptake. The effect of the hydrogel’s capacity to reuptake water after drying was examined for five consecutive cycles as shown in Fig. 9b. The results indicated that the CMC-g-(PAM-co-PAMPS) superabsorbent hydrogel demonstrated impressive water uptake efficiency, exceeding 70% after the fifth cycle, with an absorption capacity of 220 g/g. This high efficiency suggests that the hydrogel can effectively retain water even after multiple drying and rehydration cycles. Therefore, the fabricated superabsorbent hydrogel exhibited adequate mechanical properties, which are essential for maintaining structural integrity during repeated water uptake and re-drying cycles. Factually, respectable mechanical stability ensures that the hydrogel can withstand physical stresses without breaking down, allowing it to function effectively in applications such as water conservation in sandy soils. Nonetheless, the observed 30% loss in efficiency of water uptake can be attributed to several interrelated factors: (i) over repeated cycles of water uptake and drying, the mechanical integrity of the hydrogel may be compromised. The flexible cross-linking points within the hydrogel can suffer from fatigue, leading to a reduction in their ability to recover and maintain structural stability after deformation. This degradation may impact the hydrogel’s capacity to absorb water effectively in subsequent cycles; (ii) the grafting procedure used to create the hydrogel (CMC-g-(PAM-co-PAMPS)) may enhance initial water uptake but may also lead to changes in the network structure over time. As the hydrogel undergoes repeated swelling and deswelling, alterations in its porosity and connectivity can occur, which may hinder its ability to retain water efficiently; (iii) the osmotic gradient that drives water uptake can change after repeating drying cycles, making it more difficult for the hydrogel to reabsorb water.

#### Comparison with other SAHs

A comparison of the water uptake values for various reported superabsorbent hydrogels is presented in Table 2^65–75^. The results clearly indicate that the synthesized CMC-g-(PAM-co-PAMPS) SAH exhibited the highest water uptake value (313 g/g), achieved within the shortest equilibrium time (15 min) when compared to other superabsorbent hydrogels. Therefore, given the low cost and conventional applicability of the developed SAH, it holds significant potential for effective use as a water reservoir in sandy soil.

**Table 2:** Comparison of the water uptake value of the CMC-g-(PAM-co-PAMPS) SAH with other reported SAHs.

SAH	Water uptake (g/g)	Equilibrium contact time	References
Polyacrylamide-grafted gelatin	41	8 h	^65^
Carboxymethyl cellulose-graft-Itaconic acid	74	30 min	^66^
Guar gum-graft-acrylamide	80	30 min	^67^
Wheat starch crosslinked acrylic acid	120–146	24 h	^68^
Polyacrylamide grafted carboxymethyl cellulose	158	4 h	^19^
Chitosan-g-poly(acrylic acid)/attapulgite	159	8 h	^69^
Chitosan-g-poly (acrylic acid)/sodium humate	183	4 h	^70^
Pectin-g-poly(AA-co-AM) hydrogel	200	3 h	^71^
CMC/starch hydrogels	200–350	24 h	^72^
Chitosan/nano silver/acrylic acid	232	24 h	^73^
CMC-g-Poly (Sodium Acrylate)/Kaolin	261	2.5 h	^74^
Cassava starch-g-Polyacrylic acid	356	4 h	^75^
CMC-g-(PAM-co-PAMPS)	313	15 min	This study

In addition to superabsorbent hydrogels (SAHs), various materials, including resins and emulsions, have been developed to enhance water retention. For instance, a hydroxymethylcellulose sodium-g-poly(acrylic acid-co-2-acrylamido-2-methyl-1-propanesulfonic acid)/laterite (NaHMC-g-P(AA-co-AMPS)/laterite) resin has been synthesized, exhibiting a rapid water absorption rate^76^. Furthermore, a hydroxyethyl cellulose-g-poly(butyl acrylate-co-vinyl acetate) (HEC-g-P(BA-co-VAc)) emulsion has been formulated, demonstrating improved anti-leakage performance and enhanced water retention properties in soil^77^. Another category of hygroscopic materials has been developed utilizing a UIO-66-NH_2_ metal–organic framework as a functional steric cross-linker and Sa-son seed gum as a polymeric substrate. The resulting super hygroscopic hydrogels exhibit superior capabilities for water harvesting and conservation^78^. Similarly, a highly hygroscopic polymeric material featuring a three-dimensional spatial structure and controllable functionality has been designed to adsorb both liquid water and water vapor using a spherical covalent organic framework (COF-V) copolymerized with acrylic acid^79^.

### Water uptake and flow rate of water in sandy soil

**Fig. 10:**
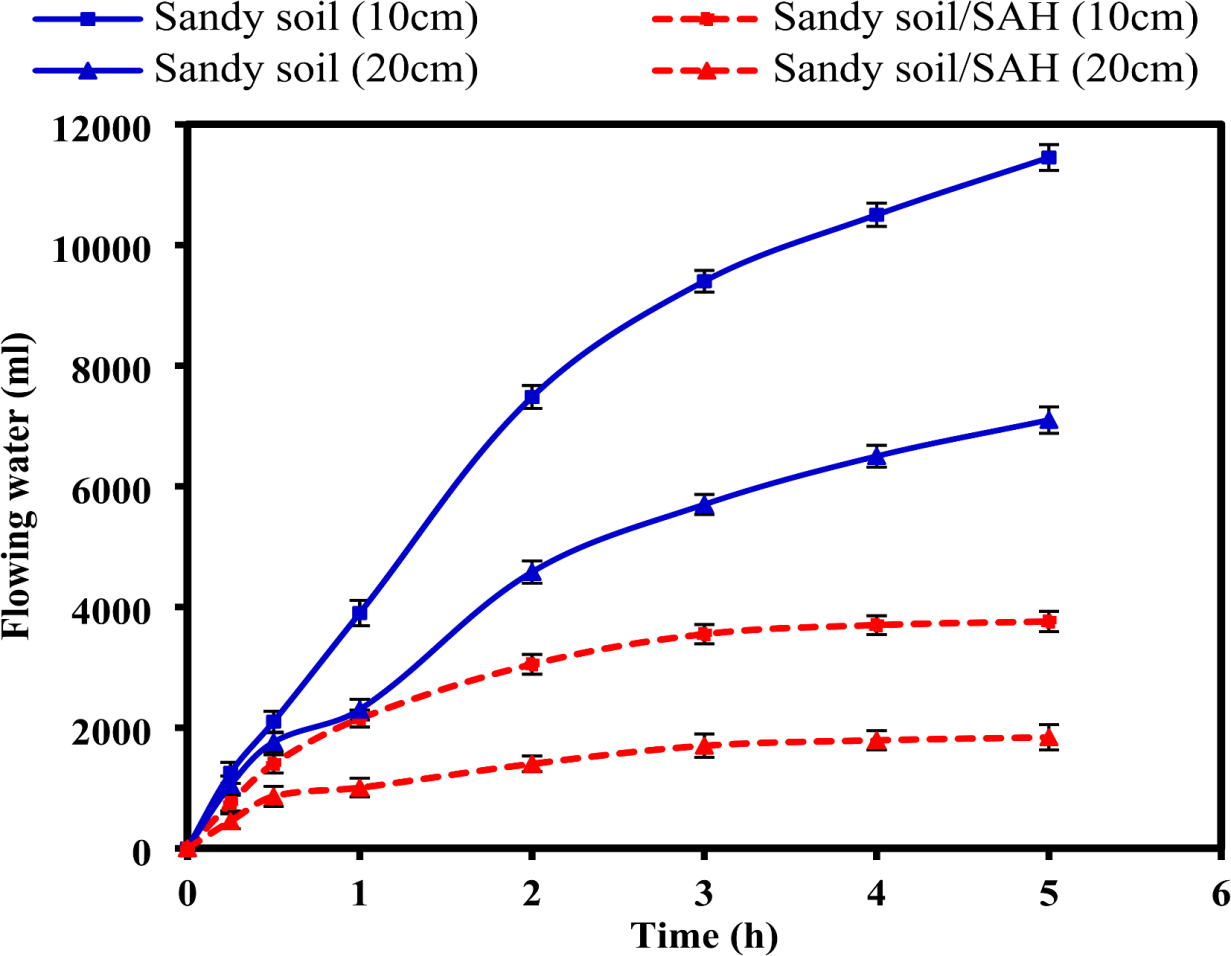
Flowing water (tap water; ml) from pure sandy soil and sandy soil/SAH using 10 and 20cm of sand layer height.

The flowing water and flow rate of water in pure sandy soil and after treatment withamendment with CMC-g-(PAM-co-PAMPS) SAH were assessed after 5has depicted in Fig. 10 and Table 3. In comparison to pure sandy soil which typically has low moisture retention due to their coarse texture, the volume of water infiltrating through sand particleswith heights 10 and 20cm was greatly reduced after adding SAH. By incorporating SAH into sandy soil, the water outflow and evaporation are minimized, and the overall water-holding capacity of the sandy soil is greatly increased. Furthermore, the results refereed that the flow rate of water wassignificantly decreasedafter the addition of SAH from 0.96 to 0.32 cm/min and from 0.61 to 0.15cm/min using 10 and 20 cm of sand layer, respectively. In fact, the primary reduction in water flow rate is attributed to the hydrogel’s rapid water absorption and swelling, which decreases pore space within the sandy soil matrix and impedes water movement^65^. This effect is further exacerbated by the increased height of the sand layer through several mechanisms: (i) the ability of the sandy soil to retain water at the top may be diminished with increasing its height due to the rapid drainage occurring in the lower layers, resulting in less water being available for flow through the entire height of the sandy soil; (ii) a longer flow path inherently increases resistance due to greater frictional forces and tortuosity; (iii) the taller sand column may result in greater compaction, particularly after SAH swelling, which further reduces soil permeability; and (iv) potential heterogeneity in SAH distribution within the longer column could amplify flow restrictions, and (v) gravitational forces may also contribute to the reduced flow rate. These combined factors likely explain the more pronounced reduction in water flow rate observed with the 20 cm sand layer compared to the 10 cm layer.

**Table 3:** Flow rate of water in sandy soil and sandy soil/SAH, and WU values of SAH in sandy soil.

Flow rate (cm/min(				WU of SAH in sandy soil (g/g)	
Sandy soil (10 cm)	Sandy soil (20 cm)	Sandy soil/SAH (10 cm)	Sandy soil/SAH (20 cm)	Sand (10 cm)	Sand (20 cm)
0.96	0.61	0.32	0.15	60	48

Likewise, the water uptake capacity of the SAH was observed to decrease from 60 to 48 g/g as the height of the sandy soil increased from 10 to 20 cm. This reduction is likely attributable to increased soil compaction at the base of the column, resulting from the overburden pressure of the soil above. This compaction can reduce the available pore spaces for water movement, thereby limiting the accessibility of water to the SAH and hindering its absorption. Additionally, the increased height may induce a more pronounced pressure gradient, influencing the rate and extent to which water is drawn into the hydrogel matrix^31,80,81^.

## Conclusion

In summary, an efficient CMC-g-(PAM-co-PAMPS) superabsorbent hydrogel has been synthesized and characterized. The SEM investigation revealed the rough surface morphology of the synthesized SAH, while the thermal degradation analysis confirmed its adequate stability at elevated temperatures. Variations in the composition ratios of the hydrogel significantly influence both the percentage add-on and the water uptake profiles. The results elucidated that the optimal grafting conditions were CMC (0.5%), co-monomers (18%), AM: AMPS (2.5:0.5), MBA (0.286%), APS (0.05%), a grafting time of 2 h, and a grafting temperature of 70 °C. Notably, the developed SAH achieved equilibrium water uptake within a mere 15 min, reaching a maximum value of 313 g/g. This optimal performance was attained with hydrogel particle sizes ranging from 250 to 500 µm, a swelling medium temperature of 40 °C, and a pH of 7. Furthermore, the SAH demonstrated superior water uptake characteristics, along with commendable mechanical stability after five repeated water uptake cycles, with a maximum efficiency reaching 70%. Additionally, the water uptake capacity of sandy soil was significantly enhanced following the incorporation of the SAH. The flow rate of water was notably reduced, recording minimum values of 0.32 and 0.15 cm/min for 10 cm and 20 cm sand layers, respectively, in contrast to the 0.96 and 0.61 cm/min observed in pure sandy soil. The results obtained suggest the potential applicability of the fabricated SAH as an effective means for water conservation in sandy soils. Despite the promising fast water uptake capabilities of the developed CMC-g-(PAM-co-PAMPS) SAH, limitations pertaining to biodegradation in sandy soil, environmental sensitivity, and cost impede their widespread adoption. Future research will focus on evaluating the SAH’s capacity for fertilizer/nutrient loading and release, assessing its biodegradability across diverse soil types and environmental conditions, and conducting comprehensive field trials to validate long-term efficacy. Addressing these limitations is crucial to unlock the full potential of the fabricated SAH for enhancing crop yields, reducing water consumption, improving nutrient delivery, and promoting sustainable agricultural practices.

## Data Availability

All data generated or analysed during this study are included in this published article.
